# Analysis of Nociceptive Information Encoded in the Temporal Discharge Patterns of Cutaneous C-Fibers

**DOI:** 10.3389/fncom.2016.00118

**Published:** 2016-11-18

**Authors:** Kyeongwon Cho, Jun Ho Jang, Sung-Phil Kim, Sang Hoon Lee, Soon-Cheol Chung, In Young Kim, Dong Pyo Jang, Sung Jun Jung

**Affiliations:** ^1^Department of Biomedical Engineering, Hanyang UniversitySeoul, South Korea; ^2^Department of Biomedical Science, Hanyang UniversitySeoul, South Korea; ^3^Department of Human and Systems Engineering, Ulsan National Institute of Science and TechnologyUlsan, South Korea; ^4^Department of Biomedical Engineering, College of Biomedical & Health Science, BK21+ Research Institute of Biomedical Engineering, Konkuk UniversityChungju, South Korea

**Keywords:** temporal decoding, temporal encoding, spike train analysis, discharge pattern, nociception

## Abstract

The generation of pain signals from primary afferent neurons is explained by a labeled-line code. However, this notion cannot apply in a simple way to cutaneous C-fibers, which carry signals from a variety of receptors that respond to various stimuli including agonist chemicals. To represent the discharge patterns of C-fibers according to different agonist chemicals, we have developed a quantitative approach using three consecutive spikes. By using this method, the generation of pain in response to chemical stimuli is shown to be dependent on the temporal aspect of the spike trains. Furthermore, under pathological conditions, gamma-aminobutyric acid resulted in pain behavior without change of spike number but with an altered discharge pattern. Our results suggest that information about the agonist chemicals may be encoded in specific temporal patterns of signals in C-fibers, and nociceptive sensation may be influenced by the extent of temporal summation originating from the temporal patterns.

## Introduction

It is generally accepted that the activation of primary afferent C-fibers by noxious stimuli leads to a sensation of pain. However, some studies have reported the lack of a pain response to activation of C-fibers, whereas others have reported an increased pain response even without an increase in the discharge rate of the C-fibers (Van Hees and Gybels, 1981; Prescott et al., 2014). Taken together, these reports suggest that a more complex neural process may exist in C-fibers, rather than the simple one-to-one relationship between sensation and receptor type according to a labeled-line code, the key coding mechanism for stimuli (Johanek et al., 2008; Pereira and Alves, 2011; Wooten et al., 2014).

Nociceptive C-fibers typically express multiple types of ion channels that respond to each agonist chemicals (Bessou and Perl, 1969; Hanack et al., 2015). For example, certain chemical nociceptors express the transient receptor potential (TRP) cation channel subfamily A1 (TRPA1), which responds to cold and mustard oil, and the subfamily V1 (TRPV1) channel responding to heat and capsaicin (Bautista et al., 2005), and their activation evokes action potentials (APs) in a single C-fiber (Han et al., 2013). However, the difference in the discharge patterns generated by the different stimuli in the C-fibers (Wooten et al., 2014) cannot be explained by the labeled-line code alone. It is assumed, therefore, that the different types of stimuli may be encoded in the discharge patterns of the C-fibers. Today, almost all workers would agree that some degree of multiple function exists in the primary afferent fibers innervating the skin, as many subpopulations have broad dynamic ranges (Kumazawa et al., 1996). Recent studies suggest that both specific function of each type of fiber, but also fiber's temporal encoding enable the generation of sensations (here, especially nociception) (Weber et al., 2013; Wooten et al., 2014). Accordingly we hypothesized that temporal encoding of spike trains from individual C-fibers may deliver key information together with combinational coding across multiple C-fibers. In a previous study, Sandkühler (1996) suggested that different responses before and after a nerve injury might be associated with the temporal patterns of spontaneous spikes, which vary according to inflammatory status. Here, we propose that activation of primary afferent C-fibers evokes nociceptive behaviors depending on temporal aspects of the spike trains, which are determined by the different chemical stimuli. Specific patterns of discharges are identified for different chemical stimuli that receptors are expressed commonly. We have analyzed these patterns by developing an analytic method that can characterize the distribution of inter-spike intervals (ISIs).

## Results

### Activation of C-fibers does not always result in nociceptive behavior

**Figure 1:**
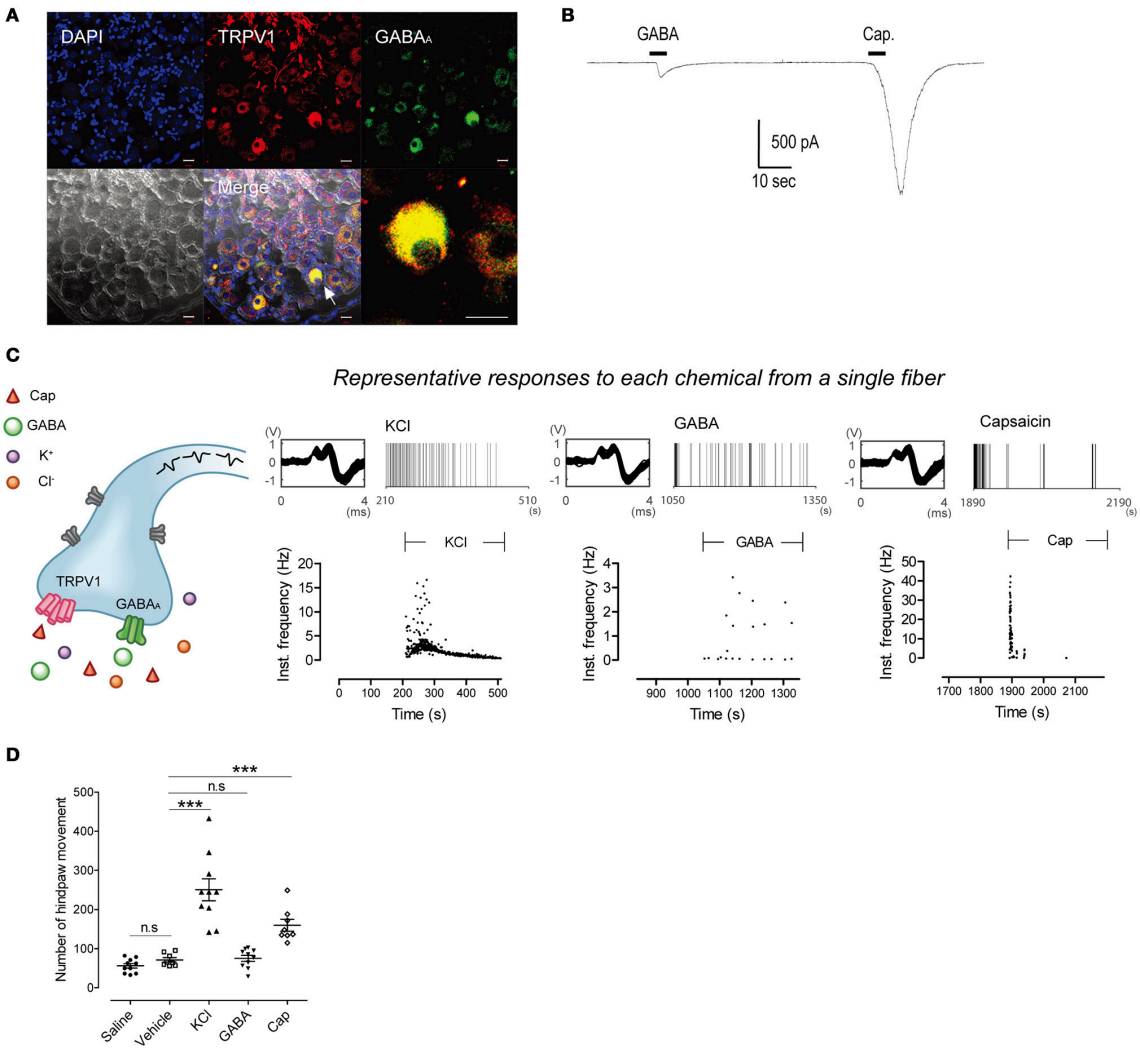
**Single fibers respond to all three chemical stimuli. (A)** Immunohistochemistry showing co-expression of GABA_A_ and TRPV1 in the same DRG neurons. Scale bar represents 20 μm. Arrow indicates GABA_A_- and TRPV1-positive neurons. **(B)** Inward currents evoked by GABA (100 μM) and capsaicin (1 μM) in small DRG neurons. **(C)** Time stamp with instantaneous frequency of the response from a representative single C-fiber under application of KCl, GABA, and capsaicin in succession. It consisted of identically shaped action potentials. **(D)** Hindpaw movements after subcutaneous injection of saline (*n* = 10), vehicle (*n* = 8), KCl (*n* = 10), GABA (*n* = 10), and capsaicin (*n* = 8). (Mann–Whitney or *t*-test based on normality, ^***^P < 0.001. Error bars represent s.e.m.).

First, we investigated whether single C-fibers could be activated by three different chemicals: Potassium chloride (KCl), gamma-aminobutyric acid (GABA), and capsaicin; due to the high intracellular chloride concentration of peripheral neurons an excitatory effect of GABA is expected. Immunohistochemistry revealed the co-expression of TRPV1 and GABA_*A*_ receptors in the some dorsal root ganglion (DRG) neurons (Figure 1A). Patch-clamp recording showed that GABA and capsaicin evoked inward currents in the same DRG neuron (Figure 1B) but that not all DRG neurons were responsive to both chemicals (7 of 28 total neurons responded for both GABA and capsaicin). In *ex vivo* recordings, 27 C-fibers were stimulated with the three chemicals in succession, with wash-out periods in between. Three fibers responded to all stimulants with identical AP shapes and sizes (Figure 1C, Supplementary Tables 1, 2).

It is known that C-fiber activation by KCl or capsaicin results in nociception. However, the sensation evoked by GABA is not reported despite the activation of C-fibers (Feltz and Rasminsky, 1974; Deschenes et al., 1976; Carlton et al., 1999). To test whether the application of GABA evoked a nociception, we performed behavioral tests for each chemical. Mouse hindpaw movements indicating pain, i.e., lifting/guarding, flinching/shaking, licking, and walking (Kawasaki-Yatsugi et al., 2012), significantly increased after subcutaneous injection of capsaicin or KCl (*Ps* < 0.001 vs. vehicle). On the other hand, GABA induced no significant increase (*P* = 0.693; Figure 1D), indicating that GABA could not induce nociception.

### The temporal discharge patterns of single C-fibers characterize different chemicals

**Figure 2:**
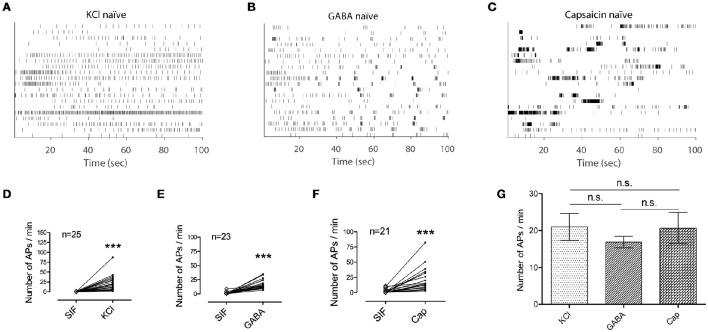
**Application of KCl, GABA, or capsaicin clearly activates primary afferents. (A–C)** Time stamp of 20 randomly selected single fibers from each group of chemical responses i.e., KCl (*n* = 25) in **(A)**, GABA (*n* = 23) in **(B)**, capsaicin (*n* = 21) in **(C)**. **(D–F)** Spike counts from each single fiber during the control period of synthetic interstitial fluid (SIF) and the corresponding responsive period to a stimulant. (Wilcoxon signed rank test, ^***^P < 0.001) **(G)** Mean of the spike counts in the evoked responses for KCl, GABA, and capsaicin are compared. (Kruskal-Wallis one-way ANOVA, n.s., not significant. Error bars represent s.e.m.).

**Figure 3:**
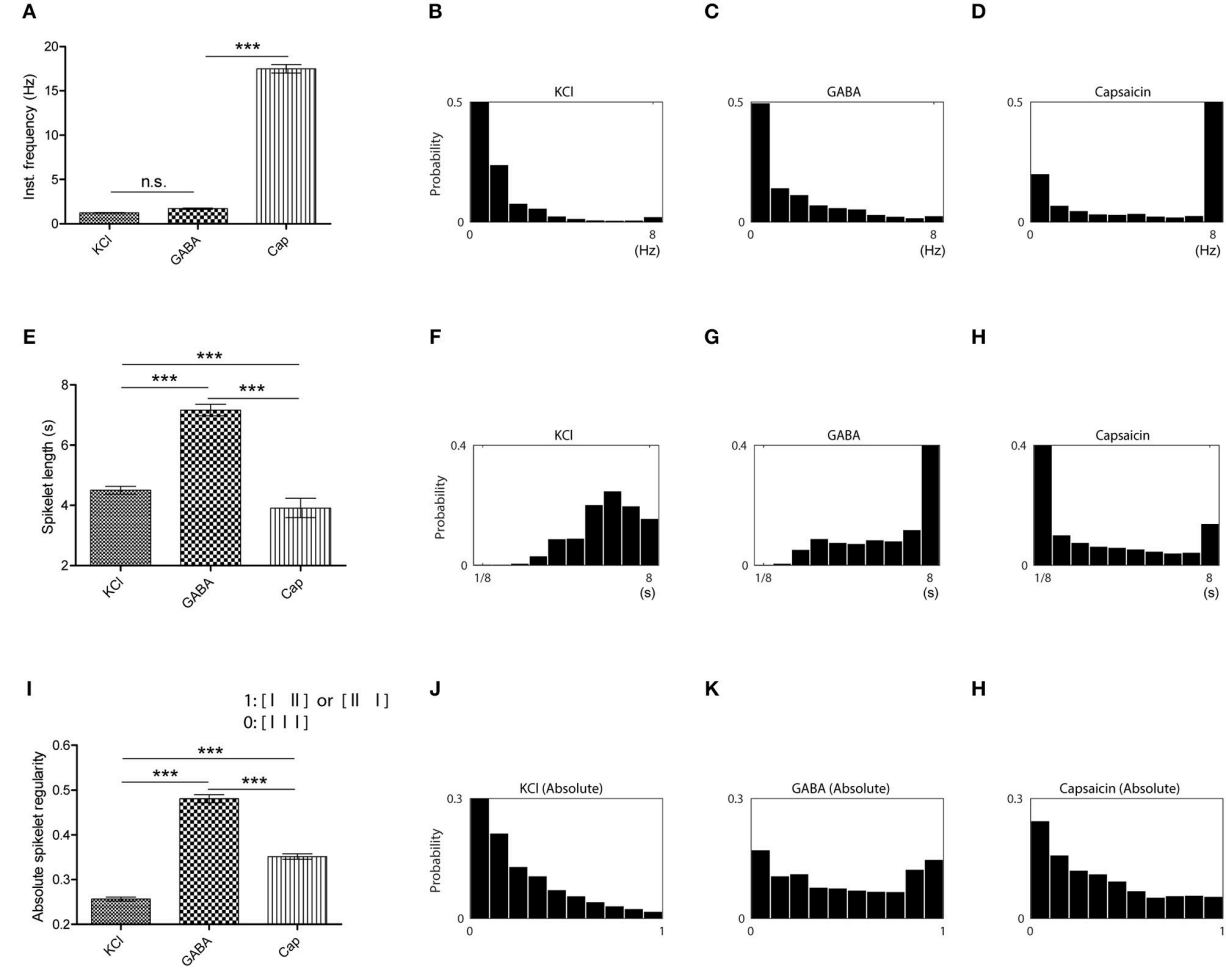
**Temporal characteristics of the different stimuli revealed in spikelet distributions. (A)** Comparison of instantaneous frequencies for KCl, GABA, and capsaicin responses. (Kruskal-Wallis one-way ANOVA followed by Dunn's test for multiple comparisons, ^***^P < 0.001, n.s., not significant.) **(B–D)** Instantaneous frequency histograms for each chemical (KCl in **B**, GABA in **C** and capsaicin in **D**). **(E)** Comparison of spikelet lengths for KCl, GABA, and capsaicin responses. (Kruskal-Wallis one-way ANOVA followed by Dunn's test for multiple comparisons, ^***^P < 0.001) **(F–H)** Spikelet length histograms for each chemical (KCl in **F**, GABA in **G** and capsaicin in **H**). **(I)** Comparison of spikelet regularity for KCl, GABA, and capsaicin responses. (Kruskal-Wallis one-way ANOVA followed by Dunn's test for multiple comparisons, ^***^P < 0.001.) **(J–L)** Spikelet regularity histogram for each chemical (KCl in **J**, GABA in **K**, and capsaicin in **L**). All the error bars represent s.e.m.

To further explore nociceptive information encoded in the activation of primary afferent C-fibers, *ex vivo* single-fiber recordings were conducted with each of the three chemicals separately (Figure 2). Analysis of the spike counts in *ex vivo* single-fiber recordings from the saphenous nerve showed that all three chemicals caused significant increases in the number of APs compared with control periods (*Ps* < 0.001; Figures 2D–F). In addition, there was no significant difference in the number of APs evoked by the three stimulants (*P* = 0.925; Figure 2G). Another measure of discharge rate is the instantaneous frequency (Lánský et al., 2004) and it is claimed that a discharge rate exceeding some threshold is associated with nociceptive behavior (Adriaensen et al., 1980). As shown in Figures 2A–C, the distribution of the instantaneous frequencies of GABA responses (mean: 1.72 Hz) did not differ from that of the KCl responses (mean: 1.25 Hz) while that of capsaicin responses (mean: 17.49 Hz) was much higher (Figures 3A–D).

As neither the number of APs nor the instantaneous frequencies could differentiate GABA responses from the other two responses, we considered the possibility that the differences might be temporally encoded in the spike trains. Visual inspection indicated that the most pronounced difference of GABA responses from the other responses lay in the repeated short bursts present in the GABA responses, we posited a fundamental unit of temporal discharge pattern to be three consecutive spikes (referred to as a spikelet hereafter), as this unit contained the minimum ISI pattern that could include both short ISIs within bursts and longer ISIs between bursts. In contrast to the results of spike counts or instantaneous frequency, GABA responses had a longer mean spikelet length (time elapsed from the first to the last spike in a spikelet) than those to the other chemicals (Figures 3E–H). The longer GABA spikelet lengths were primarily caused by a high frequency of relatively long spikelets (Figure 3G), which indicated that long and short ISIs were repeated in succession across bursts during a GABA response. To also measure asymmetry of two ISIs in a spikelet due to the intersection of long and short ISIs, we evaluated the distribution of absolute spikelet regularity (the ratio of difference between two consecutive ISIs to spikelet length) of each group and found that the regularity of GABA spikelets was significantly lower than those of KCl or capsaicin spikelets (Figures 3I–L). Both the length and the regularity of spikelets clearly distinguished the temporal pattern of GABA responses from the others, and this might be associated with the lack of nociceptive behavior from GABA.

**Figure 4:**
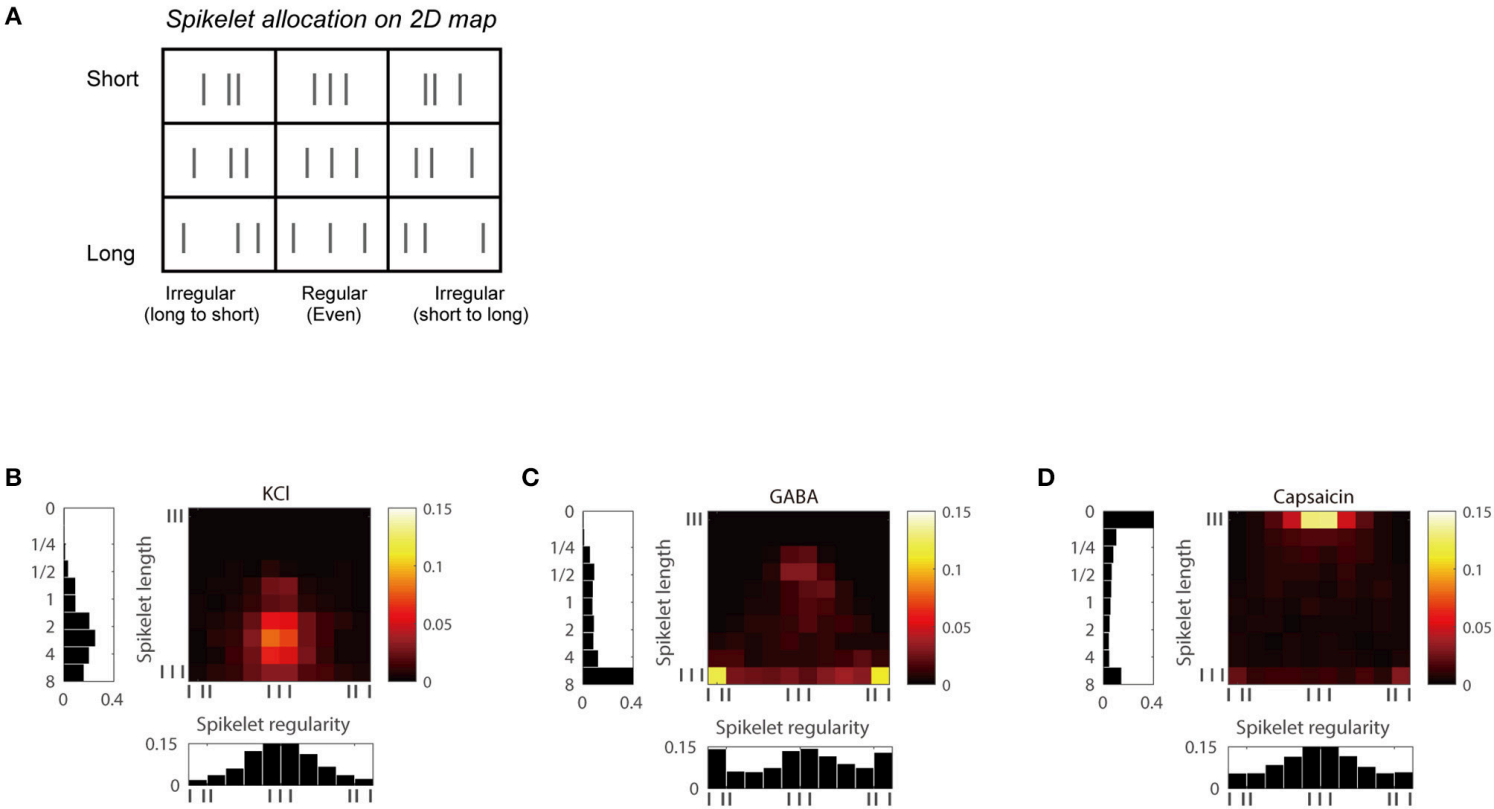
**Joint distribution maps of the length and the regularity of spikelet for each chemical. (A)** Simple 3 × 3 bin-2D map of spikelets based on spikelet length and regularity. **(B)** 2D map of KCl responses based on spikelet length (vertical axis) and regularity (horizontal axis), including histograms for each. **(C,D)** Responses of GABA in **(C)**, and capsaicin in **(D)**.

We also analyzed the joint distribution of the two temporal features, the length and the regularity of spikelets and created a two-dimensional (2D) joint distribution map of spikelets for each group (Figure 4). The maps of each group could be distinguished from the others. KCl frequently generated temporal patterns with regular spikelets of a moderate length, whereas GABA tended to generate more irregular and longer spikelets. Capsaicin tended to generate short and regular spikelets (Figures 4B–D).

The three distribution patterns were clearly different, as the k-nearest neighbor classification scheme correctly discriminated between them with classification accuracy of 79.7% (55 of 69 C-fiber responses classified correctly), which was significantly higher than chance (binomial test, *P* < 0.001; Devroye et al., 1996). In addition, nine individual maps of the three C-fiber responses obtained from the successive chemical tests in the above section were also perfectly classified into KCl, GABA, and capsaicin using a classifier based on the set of three separate spike train datasets generated by the individual chemical stimuli (Supplementary Figure 1). This indicates that C-fibers may encode information concerning the specific receptors that have been stimulated by means of unique temporal patterns.

In an attempt to estimate the effect of the temporal summation of discharge patterns on the nociceptive level, a computational model was designed using the information of spikelet length and regularity. The model was applied to the C-fiber responses with over 20 spikes during the stimulation period to estimate the nociceptive level through the temporal summation of spikelet information. As a result, the detection rate of nociception by the model was 66.7% (14 of 21) for KCl, 17.4% (4 of 23) for GABA, and 88.9% (16 of 18) for capsaicin, respectively. This result indicated that the model could properly translate C-fiber spiking patterns, represented by spikelet length and regularity, into the nociception level in response to different chemical stimuli.

### Pathological conditions inducing nociception alter the temporal discharge pattern in response to GABA

**Figure 5:**
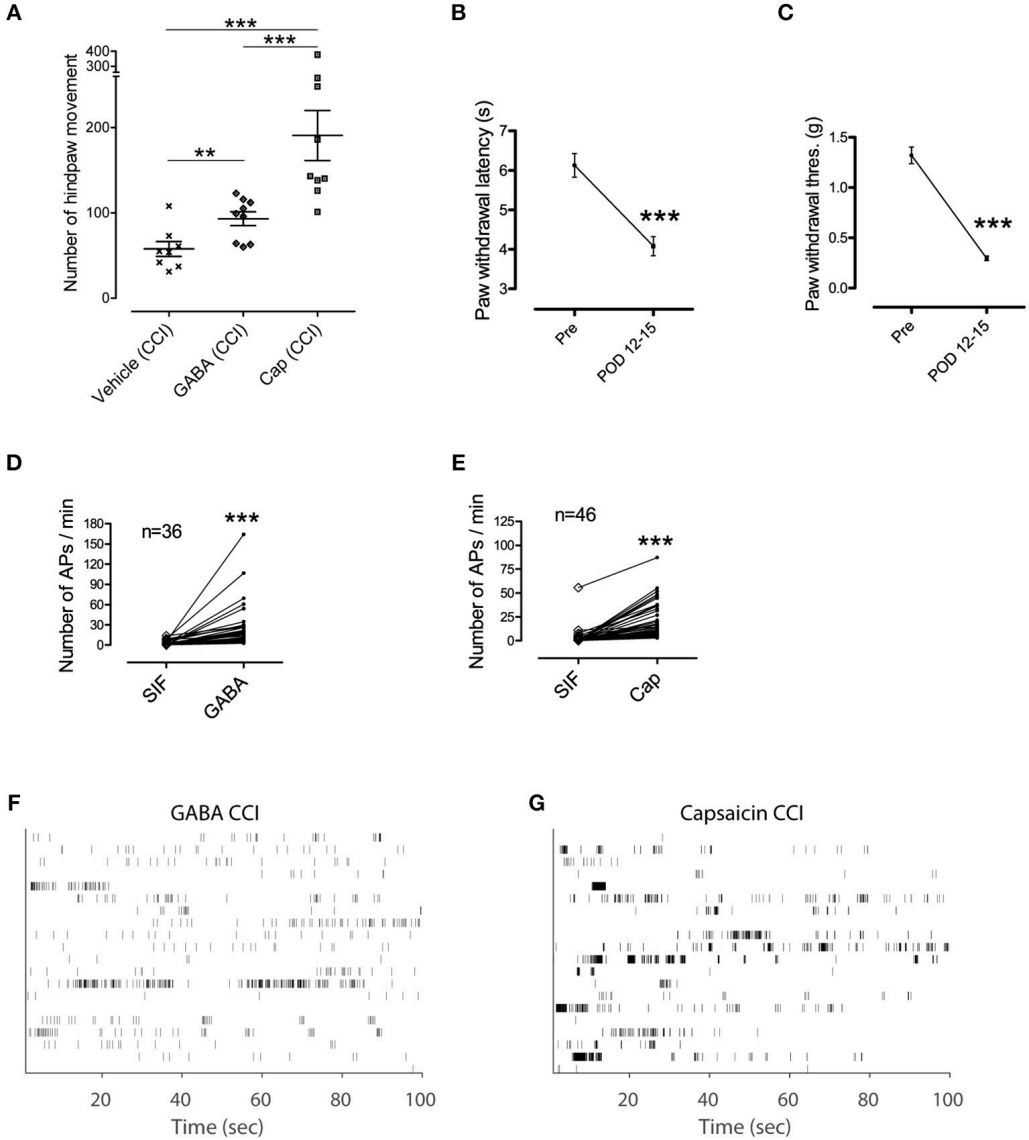
**GABA induced nociceptive behavior in pathological condition. (A)** Hindpaw movements in CCI mice after injection of vehicle (*n* = 8), GABA (*n* = 9), and capsaicin (*n* = 9). Note, that GABA induced an increase in movement. **(B,C)** Paw withdrawal test to measure pain and hypersensitivity to heat and mechanical stimuli (Wilcoxon signed rank test). Error bars represent s.e.m. **(D,E)** Spike counts in each single fiber during the control period of SIF and the corresponding responsive period to a stimulant in CCI mice. ^**^P < 0.01, ^***^P < 0.001 **(F,G)** Time stamp of 20 randomly selected single fibers in each group of chemical responses in CCI mice i.e., GABA in **(F)** and capsaicin in **(G)**.

We examined whether changes in pathological conditions could influence discharge patterns in C-fibers and subsequent nociceptive behavior (Figure 5). We conducted behavioral tests for GABA and capsaicin in mice subjected to chronic constriction injury (CCI; sciatic nerve cuffing). Compared with the controls, the number of hind paw movements were increased by both GABA and capsaicin (P < 0.01; Figure 5A), indicating that GABA induced a nociceptive behavior when exposed to a CCI.

**Figure 6:**
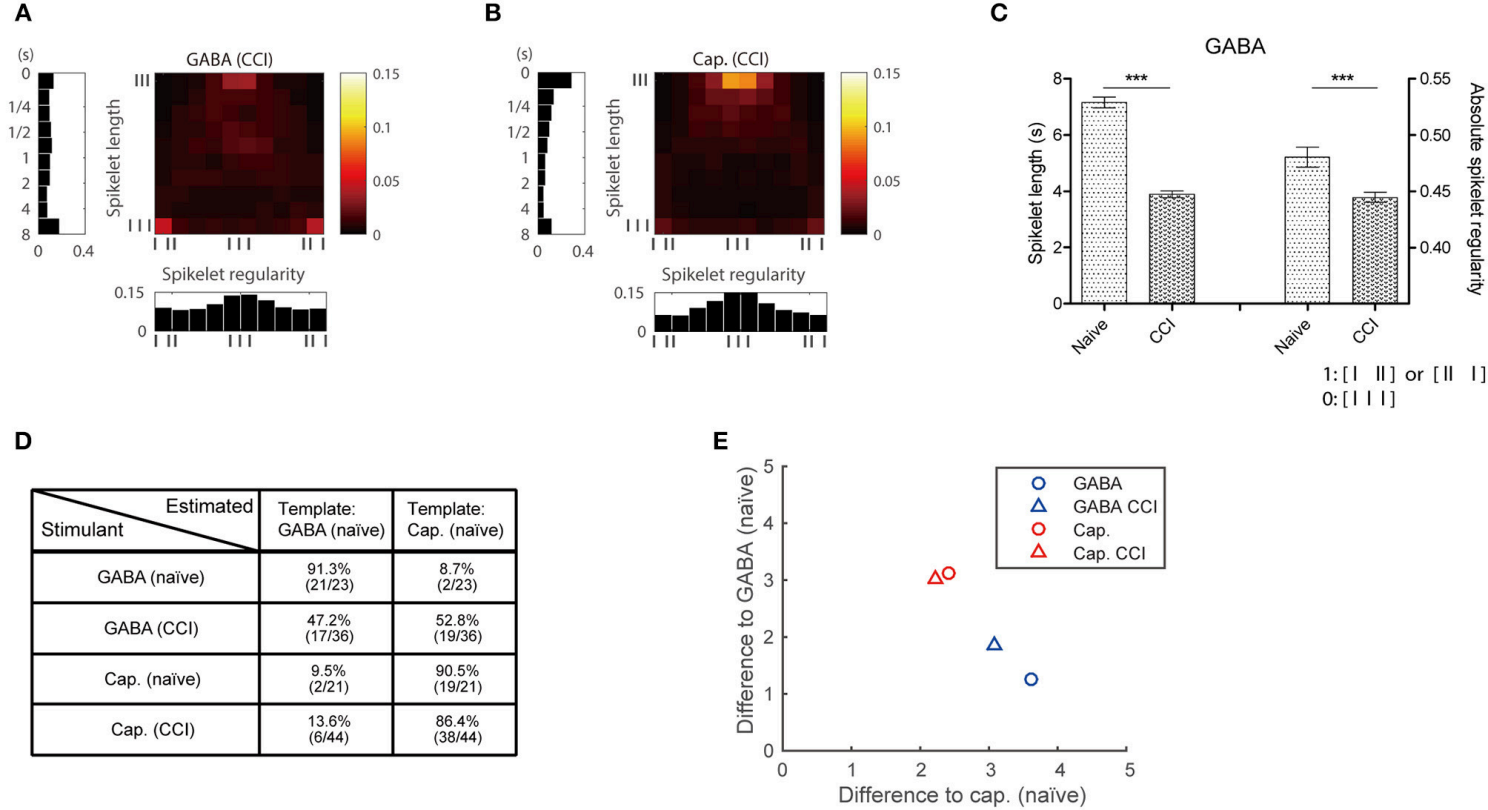
**Temporal discharge patterns in pathological condition. (A,B)** 2D map of responses in CCI mice to GABA and capsaicin, respectively. **(C)** Spikelets of GABA responses are shorter and more regular in CCI mice than naïve mice. (Mann–Whitney or *t*-test based on normality, ^***^P < 0.001) **(D)** Classification performance using KL divergence of the GABA and capsaicin patterns. **(E)** Mean differences from individual 2D maps to GABA and capsaicin 2D maps (Figures 4C,D).

The GABA and capsaicin discharge patterns were investigated using ex vivo single-fiber recordings from the sural nerves of CCI mice. First we found that the number of APs increased in response to GABA or capsaicin compared with control periods (Figures 5D,E) but there were no significant differences in the number of GABA- or capsaicin-induced APs in the CCI mice compared with the naïve mice (Supplementary Figure 2). The 2D joint distribution map for capsaicin in the CCI mice (Figure 6B) was closely akin to that in the naïve mice (Figure 4D). However, the GABA map in the CCI mice (Figure 6A) appeared to be dissimilar from that in naïve mice (Figure 4C). This suggested that the discharge pattern for a specific receptor in C-fibers could be influenced by the pathological conditions. Specifically, the short and regular spikelets, which were abundant in the capsaicin spike trains, became more frequent in the GABA spike trains of the CCI mice (Figure 6C). The abundance of the short and regular spikelets characterizing the capsaicin and CCI-induced GABA discharge patterns implied that the generation of pain might be related to the presence of high frequency continuous spikes in the afferent C-fibers. K-nearest neighbor classification analysis revealed that the binary classifier that had been trained using the GABA and capsaicin data from naïve mice had little difficulty in discriminating the novel GABA data from naïve mice but made many more errors (nearly 50% of the time) in discriminating the GABA data from the CCI mice, indicating that the GABA discharge patterns in the CCI mice were less distinguishable from the capsaicin discharge patterns (Figure 6D). Similarity analysis based on Kullback–Leibler (KL) divergence also revealed that the GABA pattern in the CCI mice was more similar to the capsaicin pattern (Figure 6E).

## Discussion

How are noxious stimuli encoded and processed to produce pain? Although the activation of C-fibers is related to nociceptive behaviors, the information may be encoded in the intervals between discharges as well as in discharge rates. Koltzenburg and Handwerker (1994) suggested that the magnitude of pain sensation is encoded by temporal summation of the nociceptive primary afferent discharge, compensating the concept of encoding by the number of APs. This study reported that the magnitude of pain increased when the repeated mechanical stimulus was more frequent, whereas the number of APs decreased. They concluded that a certain number of APs with discharge rates over the threshold is required for pain sensation. In our *ex vivo* data in naïve mice, however, spike numbers and instantaneous frequencies of spikes did not differ between the KCl and GABA groups (whereas instantaneous frequencies were significantly higher in the capsaicin group) (Figures 2G, 3A–D), indicating that additional characteristics of the temporal discharge patterns had to be explored.

To explore the neural code for nociceptive information, we applied the idea of spikelets to the ISI data acquired from the chemical-induced discharge of C-fibers. As a large value for temporal summation is achieved only if consecutive spikes have short ISIs, we supposed that temporal summation could be described well by an analysis of consecutive ISIs such as spikelets as defined in our study. Sandkühler (1996) suggested a multidimensional analysis of ISI data that represented the temporal encoding (temporal aspect of ISIs) of spontaneous discharges in spinal neurons. In principle one could take advantage of this method to visualize temporal patterns of ISIs (Debus and Sandkühler, 1996). However, this is effective only for neural activity containing at least 2000 spikes. Because of the adaptation of neural afferent activity in C-fibers, there were not sufficient numbers of spikes in our data to use Sandkuhler's method directly. Taking consecutive spikes into account, we developed spikelet analysis (based on three consecutive spikes) to characterize the temporal characteristics of spiking responses evoked by different stimuli.

Our *ex vivo* data acquired from naïve mice could be categorized into specific discharge patterns for each chemical (KCl: continuous firing; GABA: repeated short bursts or chattering; capsaicin: single or multiple bursts) using spikelet analysis. The spikelet analysis characterized the temporal patterns of the three groups by means of two parameters, namely spikelet length and regularity. Long and irregular spikelets in the GABA response developed from the start or the end of short bursts. The characteristic repeating short bursts in the GABA response that resulted in the repetition of long and short intervals might result in short temporal summation without evoking nociceptive behavior. On the other hand, short and regular spikelets were dominant in the capsaicin response and might generate longer temporal summation with pain. KCl had a greater proportion of longer spikelets than capsaicin but with little irregular ones (and long intervals), suggesting that a continuous and regular spiking pattern might be also associated with longer temporal summation resulting in nociceptive behavior (Figure 3).

GABA was not noxious in the naïve condition. However, the increase of hindpaw movement in CCI mice showed that nociceptive behavior was generated. Unlike the number of APs, the distribution of instantaneous frequencies of GABA responses was higher in CCI mice indicating an increase in the discharge rate (Supplementary Figure 3). As the spikelet length is the sum of two consecutive ISIs, the increased discharge rate is incorporated in the shorter spikelet length in Figure 6C. We measured the change of temporal components of the pattern in the CCI condition, i.e., spikelet length and regularity, and found differences in the temporal encoding of the transmitted information. According to the 2D joint distribution maps, the GABA responses in CCI mice were closer to those of capsaicin. The shift in GABA-induced temporal pattern reflected longer temporal summation, corresponding to the occurrence of pain behavior in the CCI mice.

Capsaicin-evoked responses consisted of several types of adaptation as described before (St Pierre et al., 2009). The underlying mechanism of adaptation was not considered in this study. Instead, we combined all the capsaicin responses and used them as a dataset for our analysis.

As the spikelet length and regularity revealed shorter temporal summation in GABA responses in naïve mice, the identity of a chemical stimulant can also be encoded by spikelets. Moreover, the 2D joint distribution maps of the spikelet parameters had clearly different characteristics in each condition of the fibers, indicating the possibility that a single C-fiber might encode specific biological conditions into temporal patterns. We demonstrated that nociceptive behavior was related to the temporal encoding of spike trains in primary afferent C-fibers, in which specific temporal patterns were generated according to the type of activated receptors. We also demonstrated the limitation of comparison using instantaneous frequency, and showed that nociceptive information was encoded in the temporal pattern evaluated by means of the minimum temporal unit, the spikelet. This suggests that there is a specific temporal pattern of encoding in C-fibers and the degree of temporal summation originating from the temporal patterns determines the behavioral differences. The computational model developed in the study also showed that the occurrence of nociception is estimated by discharge pattern based on spikelet. This finding corresponds to the previous studies reporting that the clustered spike trains (bursts) from the presynaptic neurons mediate the release of large dense-cored vesicles (containing neuropeptides) onto the postsynaptic membrane (Iverfeldt et al., 1989), consequently, the higher order neuron reaches the activation threshold potential. Even though the computational model does not represent an actual nociception level, it quantitatively evaluates temporal summation of spike trains that could be translated to a nociception level sensed by higher order neurons. The result of the model suggests that the nociception level might be estimated based on our spikelet analysis of discharge patterns.

In conclusion, we have expanded the concept of temporal encoding in evoked responses to sustained chemical stimuli. Our results may provide insight into the dependence of pain sensation on pathological conditions, in terms of changes in spiking patterns and receptor specificity based on a labeled-line code. The activity of multiple primary afferent C-fibers is the input for higher-order neurons (e.g., those in spinal cord); thus, the neural representation of a pain sensation may be encoded and mapped as a combination of temporal information and network processes of multiple C-fibers in a higher-order neural axis.

## Materials and methods

### Overview of animal experiments

All experiments were conducted according to the guidelines of the Animal and Plant Quarantine Agency of Korea for the care and use of laboratory animals, and the study was approved by the Institutional Animal Care and Use Committee of Hanyang University (HY-IACUC-13-037A). Male C57BL/6 mice (8 weeks old) were used throughout the study.

First, the effect of the studied chemicals on nociceptive behavior was investigated. Behavioral changes were observed in 46 mice after subcutaneous injection of one or other of the chemical stimulants, KCl (*n* = 10), GABA (*n* = 10), or capsaicin (*n* = 8), or a control, saline (*n* = 10) or vehicle (*n* = 8).

**Figure 7:**
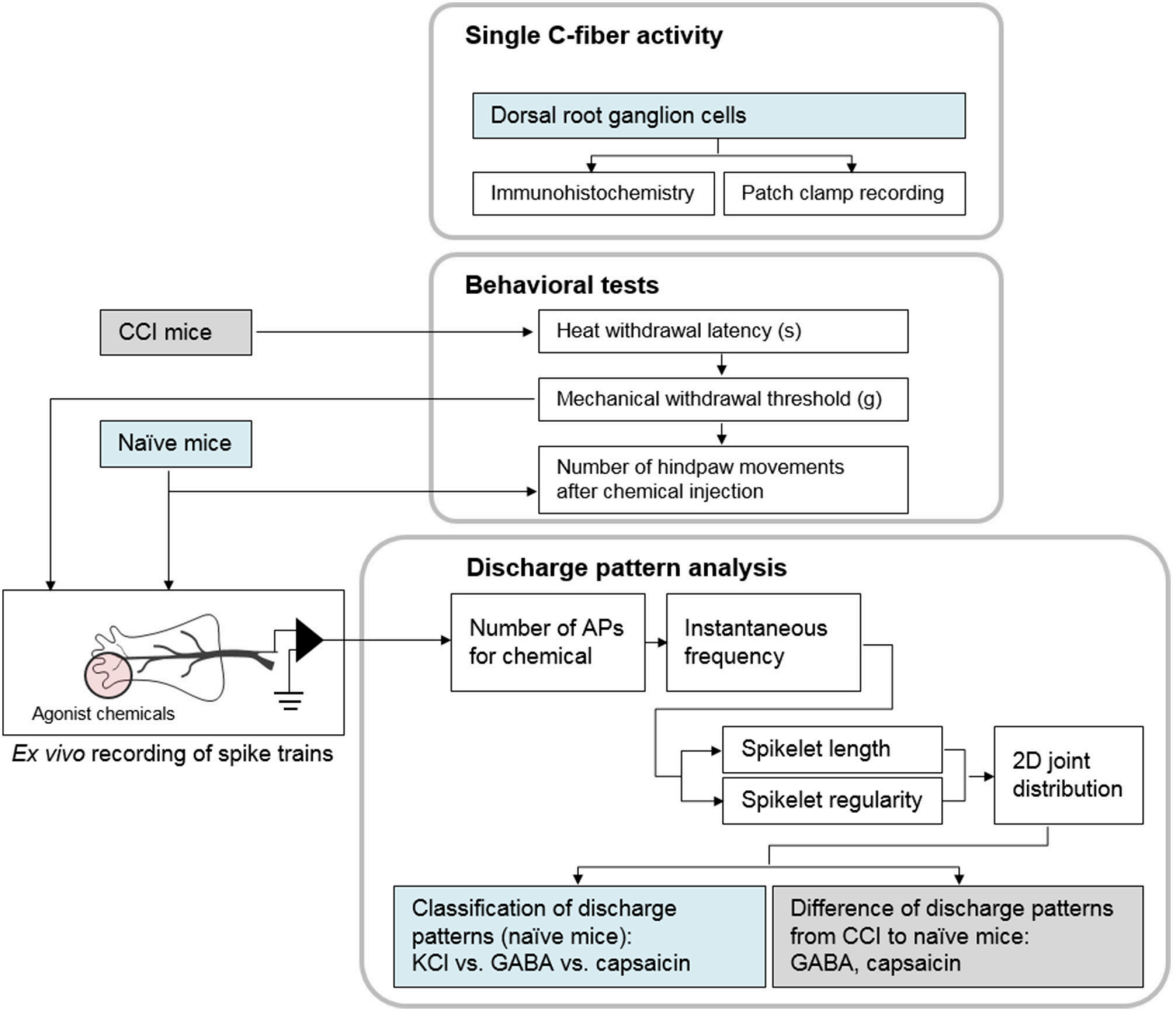
**Block diagram of the experiments**. First, we showed single C-fibers could be activated by three chemicals. Behavioral tests were followed to measure the evoked nociception after subcutaneous injection. Then, the discharge patterns in the spike trains from *ex vivo* recording were analyzed.

Next, to examine the sensitivity of single C-fibers to multiple chemicals, *ex vivo* recordings were made with 14 fibers from another group of mice. Each fiber was tested by application of all three chemicals in succession, with a wash-out period between each application. The average conduction velocity of the fibers in the sensitivity test was 0.65 ± 0.17 m/s. In addition, the effects of the three chemicals (KCl, *n* = 25; GABA, *n* = 23; capsaicin, *n* = 21) on the neural response were tested separately, with each fiber exposed to only one of the chemical stimulants. To exclude extraneous effects, the mice used for the behavioral tests were not used for *ex vivo* recording. For a pathological model, 42 mice were subjected to CCI by sciatic nerve cuffing. Hypersensitivity to mechanical and heat stimuli was verified 12–15 days after surgery. Of the 42 CCI mice 26 were used for the behavioral test (response to vehicle, *n* = 8; GABA, *n* = 9; capsaicin, *n* = 9). Fibers for *ex vivo* recording (response to GABA, *n* = 36 or capsaicin, *n* = 46) were obtained from the remaining 16 mice (Figure 7).

### Sciatic nerve cuffing model

The CCI model was used for behavioral tests based on hindpaw movement (response to stimulus to the plantar surface of the foot) as well as *ex vivo* recording from the hairy skin under pathological conditions. Surgery was performed under brief isoflurane anesthesia. The main branch of the left sciatic nerve was exposed and a cuff of PE-20 polyethylene tubing (0.38 mm internal diameter, 1.09 mm external diameter; Harvard Apparatus) of standardized length (2 mm) was applied (Benbouzid et al., 2008). The shaved skin layer was closed using sutures. Compared with baseline, the mechanical and heat thresholds were significantly reduced following sciatic nerve cuffing on postoperative days 12–15 (Wilcoxon signed rank test, *Ps* < 0.001), indicating neuropathic pain. The left sural nerve, which was affected by the CCI, was used for *ex vivo* recording.

### Behavioral studies

Mice were acclimated to testing cages containing either a stainless steel mesh (for mechanical withdrawal responses) or a heat-tempered glass floor (for spontaneous pain behavior and heat withdrawal latency) for 2 h per day for at least 5 days before testing. All behavioral tests were performed after at least 1 h acclimation on the day of the experiment.

#### Spontaneous pain behavior

Drugs were injected using gentle restraint without anesthesia. Fifty millimolars KCl, 3 mM GABA, 10 μM capsaicin, saline, or vehicle (98% synthetic interstitial fluid, SIF, mixed with 1% dimethyl sulfoxide and 1% saline) solution in a 20 μL volume was injected subcutaneously into the plantar surface of the hindpaw using an insulin syringe and a 30-gauge needle. Immediately after injection, mice were returned to the glass floor cage and 5 min video recordings were made. The number of movements of the injected limb, including lifting/guarding, flinching/shaking, licking, and walking, was determined by visual observation and considered as an indication of pain (Kawasaki-Yatsugi et al., 2012).

#### Heat withdrawal latency

The heat stimulus was a light from a projector lamp applied from underneath the glass floor onto the lateral part of the plantar surface (sural nerve territory). Before data collection, the intensity of the stimulus was adjusted so that mice withdrew after ~6 s. The latency in seconds before withdrawal was determined with a cutoff value of 12 s. The baseline values were obtained immediately before sciatic nerve injury.

#### Mechanical withdrawal responses

Von Frey filaments were applied to the lateral part of the plantar surface to estimate the 50% withdrawal threshold using the SUDO up-down method (Bonin et al., 2014). The baseline values were obtained immediately before sciatic nerve injury.

### *Ex vivo* single fiber recordings

Mice were killed using CO_2_ inhalation and the hairy skin of the hindpaw innervated by the saphenous or sural nerve was dissected after the hair on the leg was clipped. For recording from the CCI model mice, only the left sural nerve, which was impacted by the cuffing of the left sciatic nerve, was used. Attached connective tissue, muscle, and tendon were removed. The organ bath consisted of two chambers separated by an acrylic-based wall. The larger perfusion chamber was continuously superfused with a SIF (107.8 mM NaCl, 3.5 mM KCl, 0.69 mM MgSO_4_·7H_2_O, 26.2 mM NaHCO_3_, 1.67 mM NaH_2_PO_4_·2H_2_O, 9.64 mM C_6_H_11_NaO_7_, 5.55 mM glucose, 7.6 mM sucrose, 1.53 mM CaCl_2_·2H_2_O) saturated with a mixture of 95% O_2_ and 5% CO_2_ (Bretag, 1969). The temperature of the bath solution was maintained at 31 ± 1°C. After dissection, the preparation was placed with the epidermal side down. The nerves attached to the skin were drawn through one small hole to the smaller second chamber, which was filled with paraffin oil. The nerve was placed on a fixed mirror, the sheath was removed, and nerve filaments repeatedly teased apart to allow single-fiber recordings to be made using gold electrodes, one for recording and the other for reference. The reference electrode was grounded to the perfusion chamber. Signals from single nociceptive afferent fibers were recorded extracellularly with a differential amplifier (DP 311; Warner instruments). Amplified signals were sent to an oscilloscope and an audio monitor and sampled at 33 kHz, then transferred to a computer by a data acquisition system (DAP5200a; Microstar Laboratories, Inc.). APs collected on the computer were analyzed off-line using the window discrimination feature of the software (Dapsys 8; Bethel University, http://dapsys.net/). Copper blocks were connected to common ground as a reservoir of current to prevent excessive noise. The entire setup was based on a study by Zimmermann et al. (2009). The conduction velocity of the axon was determined by monopolar electrical stimulation through a low impedance electrode (CBJPL75; FHC Inc.). The supramaximal square-wave pulses (0.2–2 ms duration, 0.5 Hz) were delivered at the mechanosensitive site of a receptive field using an electrical stimulator (SD9; Grass Technologies). The distance between the receptive field and the recording electrode (conduction distance) was divided by the latency of the AP. A single C-fiber was selected on the basis of the conduction velocity (slower than 1.2 m/s); fast conducting A-fibers were excluded. The primary search strategy was mechanical stimulation by a fire-polished glass rod targeting mechanosensitive fibers.

### Temporal pattern analysis of spike trains

A histogram of instantaneous frequencies was constructed to show the empirical distribution of instantaneous frequencies for each chemical stimulus. For each chemical, all the instantaneous frequency values were divided into 10 equal-length (0.8 Hz) bins from 0–0.8 to 7.2–8 Hz. Frequency values >8 Hz were included in the last bin.

Given a spike train of *N* spikes, a spikelet *s* was defined as a set of three consecutive spikes,

(1) $$s=(t_{n},t_{n+1},t_{n+2}),$$

where *n* = 1, …, *N*−2. We characterized spikelets in terms of two simple and basic parameters: length and regularity. Spikelet length *L* was defined as the time elapsed from the first to the last spike,

(2) $$L=(t_{n+2}-t_{n}).$$

Spikelet regularity was defined as the ratio of increment of two consecutive ISIs to spikelet length,

(3) $$\Psi =2(t_{n+2}-t_{n+1})/(t_{n+2}-t_{n}).$$

Note, that spikelet regularity ranges from 0 to 2. Finally, we identified every spikelet in the spike train, allowing overlapping of two spikes between successive spikelets and calculated the length and regularity of each (i.e., there are *N*–2 spikelets in a train of *N* spikes where *N* > 2).

Having measured spikelet length and regularity from the spike train generated in response to a particular chemical, we built histograms of each parameter. The values of spikelet length were divided into 10 bins in a logarithmic scale with base 2 from 0 to 8 s: 0–0.157 s, 0.157–0.250 s, 0.250–0.397 s, 0.397–0.630 s, 0.630–1.000 s, 1.000–1.587 s, 1.587–2.520 s, 2.520–4.000 s, 4.000–6.350 s, and 6.350 s to the maximum. The maximum length was set as 8 s in our analysis, and longer lengths were included in the last bin. The absolute values of spikelet regularity, |Ψ|, were divided into 10 equal-length bins from 0–0.1, 0.1–0.2, …, 0.9–1.

#### A modeling for the estimation of nociception level

A computational model was developed to estimate a putative nociception level from the temporal integration of spikelets of an input spike train. Specifically, the model integrated the features of successive spikelets over time to estimate a nociception level and determined nociception when the estimated level exceeded a threshold. Given the *n*-th spikelet of an input spike train, our model first calculated the Gaussian radial basis function (RBF) kernels on the vector of spikelet length [*L*(*n*)] and regularity [*R*(*n*)] of the input spikelet and each of pre-determined template vectors. The template vectors included the mean vectors of spikelet length and regularity for KCl and capsaicin as well as two different vectors of spikelet length and regularity for GABA (Figures 3E,I). The two template vectors for GABA were made as either regular (Ψ = 0), and irregular (Ψ = 1) reflecting the characteristic of spikelet regularity histogram of GABA (Figure 3K). The RBF kernel width parameter, σ^2^, was empirically set to 0.1. The RBF kernel outputs, *Z*_1_(*n*), *Z*_2_(*n*), *Z*_3_(*n*), and *Z*_4_(*n*), indicated the difference of the tested spikelet from the template vectors for KCl, capsaicin, regular (Ψ = 0), and irregular (Ψ = 1) GABA responses, respectively. These four outputs were then linearly combined and fed to a hyperbolic tangent sigmoid transfer function as:

(4) $$z(n)=2/(1+{\text{e}}^{-2(Z1+Z2-Z3-Z4)})-1.$$

Note, that our model imposed positive weights (i.e., +1) on the RBF kernels for the nociceptive KCl and capsaicin [*Z*_1_(*n*) and *Z*_2_(*n*)], while imposing negative weights (i.e., −1) on those for non-nociceptive GABA [*Z*_3_(*n*) and *Z*_4_(*n*)], to estimate a nociception level. Finally, the model estimated a current nociception level, *y*(*n*), by integrating the current transfer function output [*z*(*n*)] with the previous (*n*−1)-th estimate as:

(5) $$y(n)=z(n)+\lambda^{L(n)}y(n-1).$$

A memory factor, λ, was set to a value in a range of (0, 1), describing the effect of temporal summation that would be diminished on the following spikelet. The power of λ was modeled as *L*(*n*) to implement a condition that the previous estimate would affect less with a longer spikelet. Finally, the model deemed that nociception was evoked when *y*(*n*) > θ, where θ was a threshold for the detection of nociceptive stimulations (Supplementary Figure 4).

#### Classification of discharge patterns

A joint distribution of spikelet length and regularity was created by building a 2D histogram (allocation of the spikelet to a bin on the 2D histogram map). We took into account both positive and negative values of spikelet regularity in building the 2D histogram by dividing the regularity values into 10 equal-length bins from −1 to 1 with a step of 0.2 in an ascending fashion. The counts in all the bins were divided by (*N*−2) to represent a probability distribution. We quantitatively assessed the similarity of the 2D joint distributions between different chemicals using the symmetrized KL divergence (Jeffreys, 1946). Given two probability distributions, *P* and *Q*, the KL divergence of *Q* from *P* was calculated as,

(6) $$D(P||Q)=\Sigma_{i,j}P(i,j)\text{ ln}(P(i,j)/Q(i,j))$$

with the sum over all the (*i*,*j*)-th bins, where *i* = 1st–10th bins for regularity and *j* = 1st–10th bins for length. The difference was then computed from the absolute value of the symmetrized KL divergence:

(7) Δ(P||Q)=(|D(P||Q)|+|D(Q||P)|)/2.

A larger value of Equation (7) indicated greater dissimilarity of the spikelet parameter distribution maps between chemicals. Spike trains were classified as a response to a particular chemical on the basis of the KL divergence. We first obtained a representative template of the 2D joint distribution map for each class from the training data (three classes each for KCl, GABA, and capsaicin, respectively). The representative template map was calculated by summing all bin counts per class over the 2D map followed by normalization. Next, when classifying a new spike train, we built the 2D map of the spikelet patterns and calculated the difference of it to each class template map. Lastly, the chemical of the class with the smallest difference was deemed to generate the spike train.

## Author contributions

Performed the experiments and analyzed data: KC and JJ. Provided the analysis model and the classification: SK. Conducted patch clamp recording and immunohistochemistry: SL. Conceived the project and designed the study: SC and IK. Supervised the study and edited the manuscript: DJ and SJ.

### Conflict of interest statement

The authors declare that the research was conducted in the absence of any commercial or financial relationships that could be construed as a potential conflict of interest.
