# Supplementary material for: Analysis of Nociceptive Information Encoded in the Temporal Discharge Patterns of Cutaneous C-Fibers
Source: Front Comput Neurosci. 2016 Nov 18;10:118. doi: 10.3389/fncom.2016.00118 (PMC5114282; doi:10.3389/fncom.2016.00118)
Supplement: Supplementary file 1 [file DataSheet1.pdf]

SUPPLEMENTARY EXPERIMENTAL PROCEDURES

Drug application for *ex vivo* single fiber recordings

The mechanosensitive receptive field of each fiber was isolated with a hollow metal cylinder. Silicone grease was added to prevent leakage from the bath into the receptive field. After a 3 min baseline recording, the SIF solution inside the ring was replaced with KCl (50 mM), GABA (0.3, 1, 3, or 5 mM), or capsaicin (10 μM) in a 120 μL volume, and fiber activity was recorded for 5 min. Each fiber was tested only once to avoid an effect resulting from repeated application of drugs. Fibers with a receptive field in a previously drug-treated area were also avoided for subsequent recordings. However, for sensitivity data acquisition from a single fiber (**Figure 1**), a wash-out and stabilization period of at least 5 min was used to ensure that previous residual activity had disappeared. The same protocol, including SIF (3 min) and drug application (5 min), was followed for repeated drug applications. Wash-out comprised aspiration of the drug solution and repeated pipetting of fresh SIF inside the ring. Capsaicin was applied last to avoid potential desensitization in this preparation. The fiber was considered activated by the drug if the activity of at least 2 spike/min was elicited when background activity was absent during SIF baseline recording, or if ongoing activity was more than 1 spike/min, an increase of at least 1.5 fold. Activated fibers were analyzed for data display.

Agents for stimulation

A high concentration of isotonic KCl (50 mM; 270–280 mmHg) was used as the nonspecific depolarizing stimulus. Capsaicin 1 mM stock solution was made in dimethyl sulphoxide and diluted with SIF buffer to a 10 μM working concentration. Capsaicin 10 μM was shown to elicit near-maximal AP responses from C-fibers without clear desensitization or nonspecific effects in skin nerve preparations **[1–3]**. GABA 300 mM stock was made in saline and diluted to 3 mM with SIF solution. A GABA concentration of 3 mM was selected for the experiments based on dose–response relationships within our tested concentration ranges (**Supplementary figure 4**). Vehicle was 98% SIF mixed with 1% dimethyl sulphoxide and 1% saline.

Immunohistochemistry

Mice were terminally anesthetized with isoflurane and perfused through the ascending aorta with saline followed by 4% paraformaldehyde. Dorsal root ganglia (DRGs) and spinal cord segments were removed and postfixed in the same fixative overnight. Cryostat tissue sections (10 μm) were processed for immunofluorescence as follows. Sections were blocked with 2% goat serum, and incubated overnight at 4°C with primary antibodies against $GABA_A$ (SC-7348, goat, 1:200; Santa Cruz Biotechnology) and TRPV1 (SC-28759, rabbit, 1:200; Santa Cruz Biotechnology). The primary antibodies used were validated in previous reports **[4–6]**. Sections were then incubated for 1 h at room temperature with the secondary antibodies anti-goat Alexa 488 (Life Technologies) and anti-rabbit Cy3 (Life Technologies). For double immunofluorescence, sections were incubated with a mixture of polyclonal and monoclonal primary antibodies followed by a mixture of anti-goat Alexa 488 and anti-rabbit Cy3 secondary antibodies. Sections were covered with Vectashield® mounting medium containing the fluorescent stain 4',6-diamidino-2-phenylindole (Vector Laboratories, UK) before examination at 200× under an LSM700 confocal laser scanning microscope (Carl Zeiss Microscopy GmbH). Images were captured with a high-resolution charge-coupled device photo-spot camera (Diagnostic Instruments, Inc.) and analyzed using ZEN-2013 software (Carl Zeiss Microscopy GmbH).

DRG preparation

Animals were decapitated and DRGs were rapidly removed under aseptic conditions and placed in Hank's balanced salt solution (Gibco® HBSS). DRGs were digested in 1 mg/mL collagenase A (Roche) in HBSS and 2.4 U/mL Dispase® II (Roche) in HBSS for 60 min each, followed by 8 min in 0.25% trypsin (Sigma), all at 37°C. The DRGs were then washed three times in Dulbecco's modified Eagle's medium (Gibco® DMEM) and resuspended in DMEM supplemented with 10% fetal bovine serum (FBS; Invitrogen) and 1% penicillin/streptomycin (Invitrogen). They were then mechanically dissociated using fire-polished glass pipettes, centrifuged (800 rpm, 5 min), resuspended in Neurobasal™ medium (Gibco) with B27 supplement (Invitrogen), L-glutamine and 1% penicillin/streptomycin (Invitrogen), and plated on 0.5 mg/mL poly-D-lysine (Sigma)-coated glass coverslips. Cells were maintained at 37°C in a 5% CO_2 incubator.

Whole-cell patch clamp recordings

Whole-cell patch-clamp recordings of DRG neurons were made at room temperature (25 ± 1°C) with an EPC-10 amplifier and Pulse 8.30 software (both from HEKA). For whole-cell recordings in DRG neurons, we used an external bath solution (normal Tyrode's solution: 140 mM NaCl, 5 mM KCl, 2 mM CaCl2, 1 mM MgCl2, 10 mM glucose, and 10 mM HEPES, adjusted to 305–310 mOsmol and pH 7.4 with NaOH). Patch pipettes with resistances of 3–5 MΩ were made from borosilicate glass capillaries. The pipettes were filled with a 310 mOsm internal solution containing 140 mM KCl, 5 mM EGTA, and 10 mM HEPES, pH 7.4. All drug solutions were applied to cells by local perfusion through a capillary tube (1 mm internal diameter) positioned near the cell of interest. The solution flow was driven by gravity (flow rate, 4–5 mL/min), and drugs (capsaicin and GABA) were delivered from a linear array of microcapillary tubes (1 mm internal diameter).

Statistics

SigmaPlot, Prism or MATLAB software was used for the statistical analyses. The significance of differences was tested using Mann–Whitney tests or *t*-tests based on normality, nonparametric Wilcoxon signed rank tests, nonparametric Kruskal-Wallis one way analysis of variance (ANOVA) on ranks tests, and binomial tests, as appropriate.

Supplementary references

1. Caterina, M. J., *et al.* Impaired nociception and pain sensation in mice lacking the capsaicin receptor. *Science.* **288**, 306-313 (2000).
2. McNamara, C.R., *et al.* TRPA1 mediates formalin-induced pain. *Proc. Natl. Acad. Sci. U S A.* **104**, 13525-13530 (2007).
3. Stucky, C.L., Medler, K.A. & Molliver, D.C. The P2Y agonist UTP activates cutaneous afferent fibers. *Pain.* **109**, 36-44 (2004).
4. Delgado, L.M., Vielma, A.H., Kahne, T., Palacios, A.G. & Schmachtenberg, O. *J.* The GABAergic system in the retina of neonate and adult Octodon degus, studied by immunohistochemistry and electroretinography. *Comp. Neurol.* **514**, 459-472 (2009).
5. Ferrer, I. & Puig, B. GluR2/3, NMDAepsilon1 and $GABA_A$ receptors in Creutzfeldt-Jakob disease. *Acta. Neuropathol.* **106**, 311-318 (2003).
6. Jeske, N.A., *et al.* A-kinase anchoring protein 150 mediates transient receptor potential family V type 1 sensitivity to phosphatidylinositol-4,5-bisphosphate. *J. Neurosci.* **31**, 8681-8688 (2011).

SUPPLEMENTARY FIGURE LEGENDS

Supplementary figure 1 | All the maps from each chemically evoked response of the three single fibers were well classified.

Supplementary figure 2 | Spike counts in response to GABA and capsaicin application in naïve and CCI mice. (Mann–Whitney test or t-test based on normality, $P = 0.932$ and $P = 0.\ 636$ for GABA and capsaicin, respectively). n.s., not significant. Error bars represent s.e.m.

Supplementary figure 3 | Comparison of instantaneous frequencies of GABA and capsaicin responses for naïve and CCI mice. (Mann–Whitney test or t-test based on normality, *Ps* < 0.001). Error bars represent s.e.m.

Supplementary figure 4 | Diagram of the computational model for estimation of putative nociception level.

Classification of data of single C-fibers activated by all three chemicals

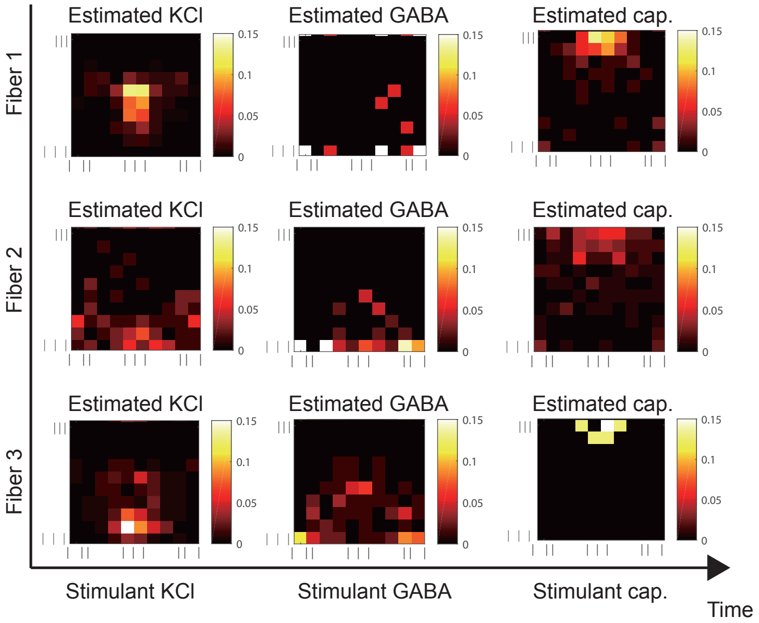

Supplementary figure 1.

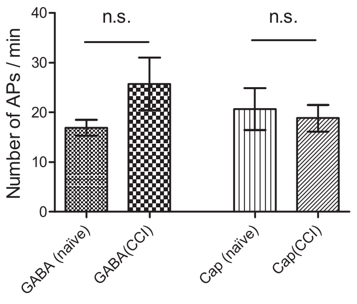

Supplementary figure 2.

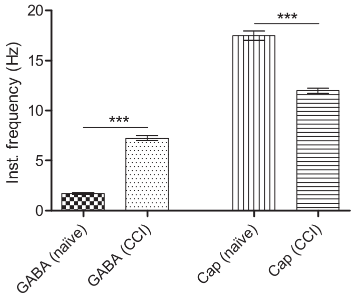

Supplementary figure 3.

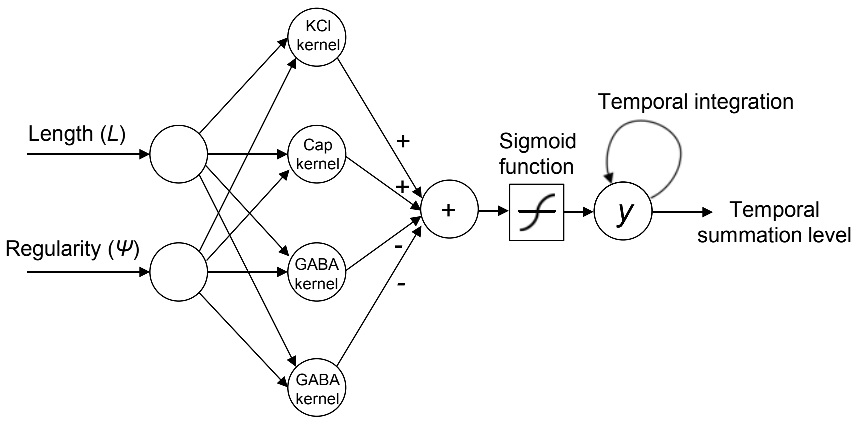

Supplementary figure 4.

SUPPLEMENTARY TABLES

Supplementary table 1 | Spike number of each C-fiber in the sensitivity test (fibers activated by all three chemicals shown in bold font).

Fiber Index \ Chemical (Time, s)	SIF (0-180)	KCl (210-510)		SIF (840-1020)	GABA (1050-1350)		SIF (1680-1860)	Cap. (1890-2190)
1	1	55	Wash-out	0	10	Wash-out	0	0
**2**	**2**	**436**		**0**	**22**		**2**	**79**
3	0	22		0	0		0	0
4	0	31		0	0		0	0
5	1	97		1	0		0	87
6	0	122		0	43		0	0
**7**	**0**	**75**		**1**	**52**		**0**	**135**
8	3	81		1	1		1	17
9	0	58		0	0		3	5
10	0	148		0	153		1	6
**11**	**2**	**149**		**0**	**65**		**0**	**10**
12	0	101		0	108		0	0
13	4	214		0	21		3	3
14	2	165		0	144		0	0

SIF: synthetic interstitial fluid. KCl: potassium chloride. GABA: gamma-aminobutyric acid. Cap.: capsaicin.

Supplementary table 2 | Fibers activated by each chemical stimulant.

KCl	GABA	Capsaicin	Number of fibers (fiber indices)
+	+	+	3 / 14 (2, 7, 11)
+		+	2 / 14 (5, 8)
+	+		6 / 14 (1, 6, 10, 12, 13, 14)
+			3 / 14 (3, 4, 9)

+: activated.
